# Bacterial communities in larger islands have reduced temporal turnover

**DOI:** 10.1038/s41396-021-00976-0

**Published:** 2021-05-03

**Authors:** Damian W. Rivett, Shorok B. Mombrikotb, Hyun S. Gweon, Thomas Bell, Christopher van der Gast

**Affiliations:** 1 Department of Natural Sciences, Manchester Metropolitan University, Manchester, UK; 2 Department of Life Sciences, Imperial College London, Ascot, UK; 3 School of Biological Sciences, University of Reading, Reading, UK; 4 Department of Life Sciences, Manchester Metropolitan University, Manchester, UK

**Keywords:** Microbial ecology, Microbial ecology

## Abstract

Patterns of species diversity provide fundamental insights into the underlying mechanisms and processes that regulate biodiversity. The species–time relationship (STR) has the potential to be one such pattern; in a comparable manner to its more extensively studied spatial analogue, the species–area relationship (SAR), which has been pivotal in the development of ecological models and theories. We sought to determine the mechanisms and processes that underpin STR patterns of temporal turnover by sampling bacterial communities within ten water-filled tree-holes on the same European beech tree through the course of a year. We took this natural model system to represent an archipelago of islands of varying sizes and with shared common immigration sources. We observed an inverse relationship between STR-derived turnover rates and island size. Further, turnover was related to island size and not island isolation within the study system as indicated by a low frequency of dispersal limitation and high homogenizing dispersal. Compared to SARs, STRs are understudied, as such, the findings from the current study should provide a renewed interest in STR-based patterns and processes.

## Introduction

A fundamental objective of ecology is to understand how biodiversity is accumulated and maintained across space and time [1]. Patterns of species diversity provide important insights into the underlying mechanisms that regulate biodiversity [1]. One such pattern, which is one of the few generalizations in ecology, is the relationship between species richness and area size [2]. The species–area relationship (SAR) has been central to the development of ecological models and theories, such as the theory of island biogeography and the unified neutral theory of biodiversity and biogeography [1, 3, 4]. Moreover, the SAR provided the foundation and impetus for the study of microbial biogeography on island and contiguous habitats [2, 5].

In contrast, the manner in which species richness changes with time has received less attention than that of the SAR. The SAR is well described with the power law equation *S* = *cA*^*z*^, where *S* is the number of observed species in area *A*, *c* is an empirically derived taxon- and location-specific constant and *z* is the slope of the line or spatial scaling exponent [4]. Increasing values of *z* can be taken as greater rates of turnover or accumulation with area [1]. Originally proposed by Preston [6], the species-time relationship (STR) describes how the observed species richness of a community in a fixed area increases with the length of time over which the community is monitored [6, 7]. The species–area power law can be modified to describe the relationship between species richness and time, *T*. For clarity, the scaling exponent is changed from *z* to *w*, so that the STR power law becomes *S* = *cT*^*w*^ [7, 8].

The steepness of the STR slope (*w*) can be used to describe how local communities turnover in time [8–10]. This is analogous to the SAR slope (*z*) that describes how local assemblages differ in space [1]. A meta-analysis of STRs from ecological communities from a wide variety of eukaryotic organisms (including algae, zooplankton, invertebrates insects, fish, birds, plants mammals, and corals), and from a range of aquatic and terrestrial ecosystems, found a remarkable degree of regularity in STR scaling exponents. Values of *w* typically ranged between 0.2 and 0.4, with a minimum of 0.10 and maximum of 0.51 [10]. In agreement, meta-analyses of a wide range of microbial STRs found temporal scaling exponents were also typically within this range [8, 9]. It is also notable that scaling exponents for animal, plant, and microbial communities are also similar despite differing methods used to construct STRs (e.g., cumulative moving window [8] or every possible window [10] approaches), varying lengths in time series, and diverse sampling methods and depths (from classical ecological census techniques to high-throughput sequencing methods) [9, 11]. Interestingly, it has been demonstrated that STRs can be used as informative indicators of biological integrity and ecosystem health following anthropogenic disturbance, including decreasing temporal scaling exponents in response to increasing selection pressure and increasing pollutant concentration in environmental perturbations [8, 12, 13].

Crucially, the ecological processes and mechanisms that underpin the empirical STR patterns of temporal turnover remain to be determined [14]. To address this knowledge gap, we sampled bacterial communities within ten water-filled tree-holes on the same tree through the course of a year. We took this natural model system to represent an archipelago of islands of varying habitat size and with shared common immigration sources. Rainwater accumulates in bark-lined pans formed by buttressing at the base of large European beech trees (*Fagus sylvatica*) to form small but often permanent bodies of water [2]. Each of these islands houses a micro-ecosystem that derives its nutrients and energy from decomposing leaf litter [15]. Vitally, for the current study, tree-holes have been used previously as tractable experimental microcosms in microbial ecology to address questions of fundamental ecological importance, e.g., [2, 12, 16, 17]. In addition, we also investigated the effects of using differing approaches to construct STRs, and implications sampling depth and time-series length had on the resulting STRs and temporal scaling exponents (*w*), providing recommendations for further study.

## Materials and methods

### Sampling procedure

All samples were taken from a single mature European Beech tree (*Fagus sylvatica*) located in the grounds of Silwood Park, Ascot, UK (51° 24’ 29.52”, −0° 38’ 42.72”). A number of tree-holes were identified within the root system, of which ten were deemed suitable (i.e., not connected, and able to hold water and detritus) for inclusion in the survey. Samples were collected from November 2014 to November 2015. From each tree-hole, surface area of the tree-holes was determined as an ellipsoid, by measurement of the perpendicular radii. The volume of all tree-holes was measured by homogenizing the water and sediment contained within the tree-holes and siphoning the liquid into measuring cylinders, as previously described [2]. Samples of ≈5 g of the homogenized slurry were taken and frozen for molecular analysis at −80 °C. Metadata, including dates and days of sampling, sample durations, tree-hole surface areas, and volumes for each tree-hole are available at figshare.com under 10.6084/m9.figshare.10320713.v1.

### Sequencing

Nucleic acids were extracted from the samples using the protocol previously described [18]. Quantification of total nucleic acids was approximated using a Take3 Micro-Volume plate (BioTek, Swindon, UK) and Synergy2 spectrophotometer (BioTek). Samples were diluted to 10 ng/μL using Starlet Micropipetting liquid handling system (Hamilton Robotics, Reno, NV, USA) and custom protocols. The V4 region (~250 bp) of the bacterial 16S rRNA genes were amplified using the dual indexing PCR protocol described in [19], allowing 364 samples to be uniquely indexed. Briefly, 1 μL nucleic acids (~10 ng) were used as the template in a 50 μL reaction volume consisting of 0.5 μL Q5 High Fidelity Taq (2000 unit ml^−1^), 10 μL 10X reaction buffer, 10 μL GC enhancer, 1 μL 10 mM dNTP, 22.5 μL molecular grade water and 5 μL of dual indexed primer (0.125 μM of each forward and reverse primer). Parameters for the PCR were as follows; initial denaturing at 95 °C for 2 min followed by 25 cycles of 15 s at 95 °C, 15 s at 55 °C and 30 s at 72 °C, with a final extension time of 10 min at 72 °C. Amplifications were confirmed on 1% agarose gel stained with GelRed (Biotinium, Inc. Fremont, CA, USA). Amplicons were normalized to up to 25 ng per sample using SequalPrep Normalization Plate Kit (Thermo Fisher Scientific, Loughborough, UK) and libraries were pooled per plate (up to 96 samples per plate). The four libraries were quantified using Qubit High Sensitivity (Thermo Fisher Scientific) and pooled in equal concentration. 400 pM library and 40 pM PhiX (Illumina, Inc., San Diego, CA, USA) control were prepared and denatured with 2 μL 2 N NaOH (Sigma-Aldrich, Gillingham, UK) for 5 min at room temperature then neutralized with 2 μL 2 N HCl (Sigma-Aldrich). An 8 pM library with 10% PhiX control was created with chilled HT1 buffer and loaded into the V3 chemistry MiSeq cartridge (Illumina, Inc.) to achieve 2 x 300bp sequencing reads.

### Sequence analysis

Sequenced paired-end reads were joined using PEAR [20], quality filtered using FASTX tools (Hannon, http://hannonlab.cshl.edu), presence of PhiX and adapters were checked and removed with BBTools (jgi.doe.gov/data-and-tools/bbtools), and chimeras were identified and removed with VSEARH_UCHIME_REF [21] using Greengenes Release 13_5 (97%) [22]. Singletons were removed and the resulting sequences were clustered into operational taxonomic units (OTUs) with VSEARCH_CLUSTER_FAST [21] at 97% sequence identity [23]. Representative sequences for each OTU were taxonomically assigned by RDP Classifier with the bootstrap threshold of 0.8 or greater (Wang et al, 2007) using Greengenes Release 13_5 [22] as the reference. Resultant OTUs were combined to create phylotypes, associated at the 97% identity similarity cut-off, which roughly corresponds to a species/genus level [23]. Bioinformatics code is provided in the Supplemental materials. The raw sequence data reported in this study have been deposited in the European Nucleotide Archive under study accession number PRJEB35208. Metadata, including sample identifications relating to the sequences accessions, is available at figshare.com under 10.6084/m9.figshare.10320713.v1.

### Statistical analyses

Three differing methods were used to construct STRs; termed here as the ‘moving window’, ‘cumulative moving window’ [8], and ‘every possible window’ [10] approaches. Note, in all approaches, new taxa were defined as the number of taxa present in the last sample of a window, but not observed in the first sample or cumulative samples as a window was moved sequentially along a time series. For the ‘moving window’ (MW) approach, adjoining sample time points were taken pairwise moving along the time series, with the richness of the first sample added to the number of new taxa found in the second. For example, in a 20-time point time series, richness in sample 1 is added to the new taxa observed in 2, then 2 and 3, 3 and 4, 4 and 5, etc., up to 19 and 20. The ‘cumulative moving window’ (CMW) differed from the ‘moving window’ approach in that only the first appearance of each bacterial taxon was used, despite that some taxa emerged and disappeared multiple times across a time series within a given tree-hole [8]. Specifically, taxa richness in sample 1 is added to the number of new taxa in sample 2, then (1, 2) + new taxa in 3, (1, 2, 3) + 4, (1, 2, 3, 4) + 5, etc., up to (1, 2, 3 …. 17, 18, 19) + new taxa in 20. For the ‘every possible window’ (EPW) approach, change in taxa richness was determined for every possible window of all potential time spans, and the mean new species value recorded at each sampling point. Hence, the 20-time point time series example is broken down into 20-time point windows, 19 two-time point windows, 18 three-time point windows, etc. [10]. This results in taxa richness being recorded for each sample in the 20 single time point windows, after which, taxa richness of the first sample (or cumulative samples) was added to the number of new taxa in the subsequent sample of that sequence as the window moves through the time series. Hence, for the two time point/paired windows: richness in sample 1 was added to the number of new taxa present in sample 2, the window then moved onto samples 2 and 3, 3 and 4, and so on up to samples 19 and 20. For the three time point windows: the taxa richness in samples 1 and 2 have the new taxa in sample 3 added, then (2, 3) + 4, (3, 4) + 5, etc., up to (18, 19) + 20. For the four time point windows: (1, 2, 3) + new taxa in 4, then (2, 3, 4) + 5, (3, 4, 5) + 6, etc., up to (17, 18, 19) + 20. Window size continued to increase sequentially in that manner until arriving at the final twenty point window, e.g. (1, 2, 3 …. 17, 18, 19) + new taxa in 20. These values were then averaged within each time span prior to plotting the STR [10]. All STRs were constructed and plotted in Microsoft Excel (Microsoft Corporation, Redmond, Washington, USA). All regression analyses, coefficients of determination (*R*^2^), degrees of freedom, *F*-statistics, and significance (*P*) were calculated using XLSTAT v2018.1 (Addinsoft, Paris, France).

To test to what extent temporal turnover within each of the tree-hole communities were accounted for by Vellend’s rationalized ecological processes [14], local communities were compared using a Monte Carlo procedure (1000 randomizations) to determine whether any two communities were more or less similar than expected by chance using the Raup and Crick probability-based index of similarity (*S*_RC_) [24]. For each tree-hole, the ‘regional’ species pool was defined as all species that occurred through the time series for all tree-holes. The *S*_RC_ probability-based index, which is independent of sample size and based on presence–absence data, was rescaled to range from 1 to −1 [24], but, contrary to Chase et al., maintained as an intuitive measure of similarity and not dissimilarity. Pairwise *S*_RC_ indices of ≥0.95 and ≤−0.95 are significantly similar or dissimilar, respectively, than expected by chance, and *S*_RC_ indices between 0.95 and −0.95 indicate similarity no greater than expected by chance [24]. Recently, this has been extended to quantify which ecological processes shape differences between local communities [25]. When *S*_RC_ is used as a similarity index, values near 1 (0.95–1) indicate homogenizing dispersal, values near −1 (−0.95 to −1) indicate dispersal limitation (selection), and values between 0.95 and −0.95 indicate drift. *S*_RC_ indices were calculated using PAST v3.25 (https://www.nhm.uio.no/english/research/infrastructure/past/).

## Results and discussion

**Fig. 1:**
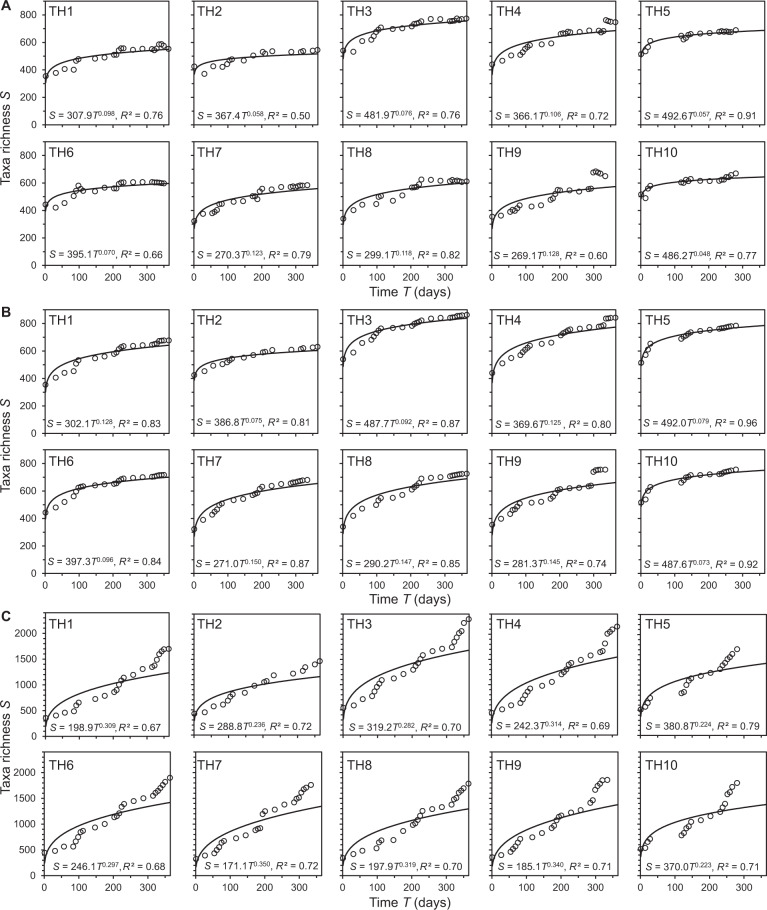
Species-time relationships for the tree-hole bacterial communities. **A**, **B**, and **C** represent species–time relationships (STR) constructed using every possible window, cumulative moving window, and moving window approaches, respectively. Given in each instance is the tree-hole number (TH1–TH10) and the STR power law equation. All STRs were significant (*P* < 0.001). Full regression summary statistics are provided in Table S1.

**Fig. 2:**
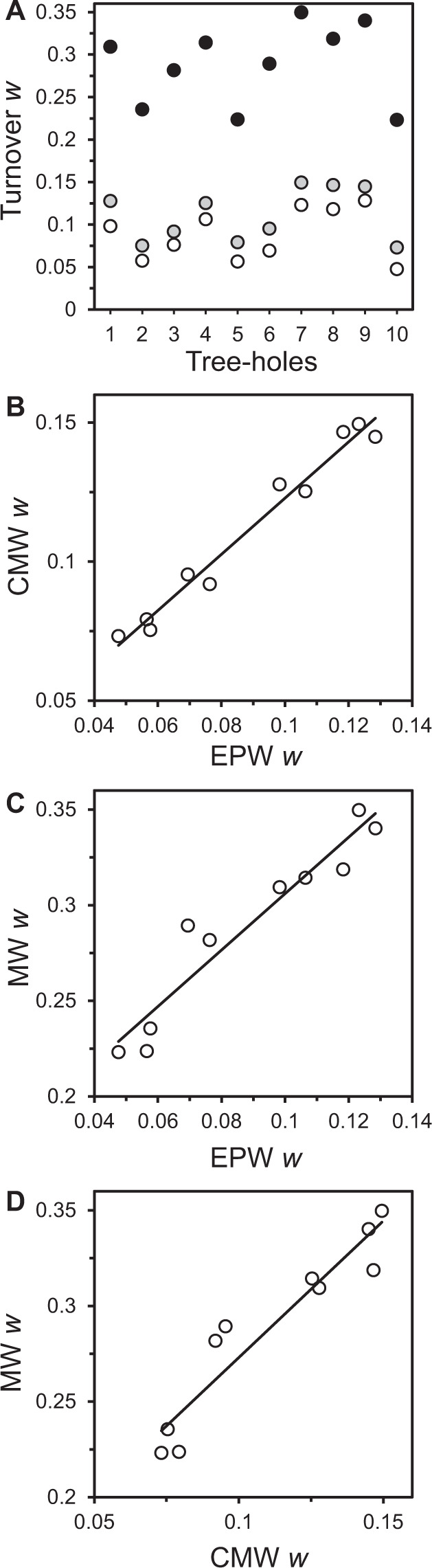
Comparison of temporal scaling exponents (*w*) between STR construction methods. **A***w* values plotted by tree-hole and STR construction method: Every possible window (EPW) approach, unfilled circles; cumulative moving window (CMW), grey circles; and moving window (MW), black circles. Relationships between (**B**) CMW and EPW, (**C**) MW and EPW, and (**D**) MW and CMW *w*-derived exponents. One-way ANOVA summary statistics (**A**): EPW vs CMW, *F*_1,18_ = 2.74, *R*^2^ = 0.13, *P* = 0.115; EPW vs MW, *F*_1,18_ = 87.1, *R*^2^ = 0.88, *P* < 0.0001; and CMW vs MW, *F*_1,18_ = 99.9, *R*^2^ = 0.85, *P* < 0.0001. Regression statistics (**C**–**D**): CMW and EPW, *R*^2^ = 0.97, *F*_1,8_ = 286.7, *P* < 0.0001; MW and EPW, *R*^2^ = 0.91, *F*_1,8_ = 82.7, *P* < 0.0001; MW and CMW, *R*^2^ = 0.90, *F*_1,8_ = 71.4, *P* < 0.0001.

The underpinning method employed to construct a STR may affect the shape, scaling exponent (*w*), and fit of the STR power function. Here we used three differing approaches to construct STRs; including, what we term in this study, the ‘every possible window’ (EPW) [10], ‘cumulative moving window’ (CMW) [8], and ‘moving window’ (MW) approaches. The differences in each method are extensively detailed in the Material and Methods. The STRs for the bacterial communities within each of the tree-hole islands were plotted, of which all relationships were significant (Fig. 1 and Table S1). Overall, the resulting STR power law exponents (*w*) were found to range from 0.048 to 0.350 (Fig. 1) and were typically within the exponent ranges observed from meta-analyses of STRs for a wide range of animals, plants, and microbial communities [9–11]. However, these values varied by the approach used to construct STRs (Fig. 2A). The EPW based *w* values ranged from 0.048 to 0.128, with a mean *w* of 0.088 ± 0.029 (mean ± SD). The CMW *w* values ranged from 0.073 to 0.150, with a mean *w* = 0.111 ± 0.029. Whereas, the MW minimum and maximum *w* values were 0.223 ± 0.350, with a mean of 0.289 ± 0.044 (Fig. 2A). The EPW and CMW *w* values were significantly lower than the MW *w* values (Fig. 2A). However, they were not significantly different from each other, despite that EPW values were uniformly lower (Fig. 2A).

A key difference between the EPW and CMW approaches when compared to the MW is the former only used the first appearance of each bacterial taxon, even though taxa can emerge and disappear multiple times across a time series for a given tree-hole. Conversely, the MW approach does incorporate multiple immigrations and extinctions of the same taxa through time, which would be anticipated in a time series of this study’s extent. This would result in higher turnover, as observed here, and could therefore provide rational explanation for the higher residuals observed towards the latter portions of the MW STRs (Fig. 1C). However, to ascertain whether higher residuals are a general phenomenon or specific to the study system, and hence whether a power function is best to describe an empirical STR, would require wider comparative testing of the STR construction methods across a broad range of microbial communities from different habitats. Regardless of the STR construction method used, variation in *w* values across the tree-holes mirrored each other (Fig. 2A); all were significant and highly correlated between each approach (Fig. 2C–D). Previously, a fundamental reason for only using the first appearance of each taxon was due to the detection limits within the underpinning method used to survey microbial communities. For example, earlier work on STRs used fingerprinting methods, such as Denaturing Gradient Gel Electrophoresis, which were known to be affected by detection thresholds e.g., [8, 11–13]. In which, the disappearance and reappearance of a bacterial taxon could have been due to dropping below the detection threshold as opposed to going locally extinct. Whereas, this is now less of an issue with the high-throughput sequencing approaches and therefore a higher degree of confidence can be taken when assessing local immigration and extinction.

Next, we investigated the possible influence of time series length and sampling depth on the resulting temporal scaling exponents. Within the current study, sampling duration varied from a minimum of 280 days to a maximum of 364 days, mean = 339.8 ± 32 days. Likewise, mean sequencing depth varied across the tree-holes, with a minimum of 33893 and maximum of 47283 sequence reads, and mean of 41521.3 ± 4147.7 sequence reads. Subsequent analysis revealed STRs, regardless of construction method, were not related to variation in study duration or sequencing depth (Figure S1: EPW with time, *R*^2^ = 0.16, *F*_1,18_ = 1.58, *P* = 0.245; CMW with time, *R*^2^ = 0.14, *F*_1,18_ = 1.34, *P* = 0.281; and MW with time, *R*^2^ = 0.28, *F*_1,18_ = 3.12, *P* = 0.115; EPW with mean sequence depth, *R*^2^ = 0.001, *F*_1,18_ = 0.001, *P* = 0.953; CMW with mean sequence depth, *R*^2^ = 0.01, *F*_1,18_ = 0.001, *P* = 0.948 and MW with mean sequence depth, *R*^2^ = 0.002, *F*_1,18_ = 0.002, *P* = 0.902). Therefore, this would indicate that any influence based on sampling was negligible, as has been observed previously [9]. Although there is an observed regularity in the STR exponents, found here and more broadly, there remains variability within that range. It has been previously posited that finding patterns within that variability could provide a better understanding of the processes underpinning STRs, and hence temporal turnover within ecological communities [10].

**Fig. 3:**
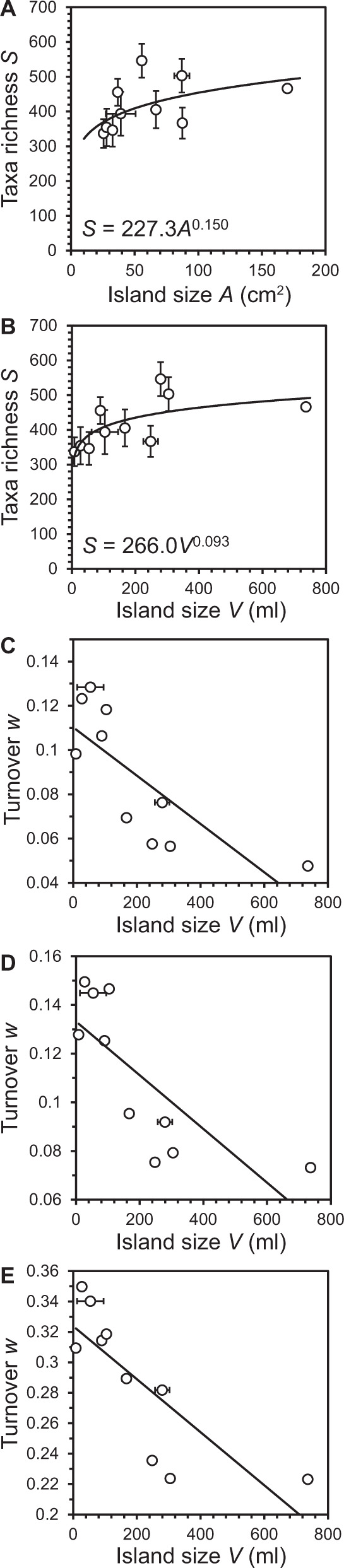
Island size relationships with taxa richness and turnover. Given are taxa richness relationships with (**A**) tree-hole surface area, *A*, and (**B**) tree-hole volume, *V*, as measures of island size. Also given, in each instance, are the power law equations for the species–area and species–volume relationships. Regression summary statistics: (**A**) *R*^2^ = 0.30, *F*_1,8_ = 3.4, *P* = 0.104; and (**B**) *R*^2^ = 0.52, *F*_1,8_ = 8.8, *P* = 0.018. **C**–**E** Relationship between taxa turnover (*w*) and island size (*V*) for different STR construction approaches: (**C**) *w* values derived from every possible window approach (*R*^2^ = 0.60, *F*_1,8_ = 12.2, *P* = 0.008); (**D**) cumulative moving window approach (*R*^2^ = 0.59, *F*_1,8_ = 11.1, *P* = 0.01); and (**E**) moving window approach (*R*^2^ = 0.64, *F*_1,8_ = 14.4, *P* = 0.005). Vertical and horizontal error bars represent standard deviation of the mean of richness and island size, respectively, over census time.

For island-based communities, theory predicts that turnover rates should be inversely related to island size and to island isolation [4, 26]. For the latter, we presume that isolation from source of immigration for the tree-hole islands was approximately equivalent and hence would have limited effect on turnover rates; as the main routes of bacterial immigration would be via falling leaf matter predominately from the host tree, and rainwater running down the trunk of the tree from the canopy above. Before a relationship between turnover and island size could be tested, the best measure of island size had to be determined. Although island area has been the traditional measure [1, 4], volume has been found to be a better measure of island size in microbial studies based in aquatic habitats [2, 27, 28]. We tested this by plotting the SAR and species–volume relationship across the tree-hole communities, accounting for variation in area, volume, and richness over time, and found volume to be the better predictor (Fig. 3A&B). Using volume as the measure of island size, a significant inverse relationship with temporal turnover was observed (Fig. 3C). In agreement with theoretical predictions for island biogeography, we found that smaller islands had lower taxa richness, but, with higher turnover, whereas larger islands had greater richness with reduced turnover over time (Fig. 3B&C).

There is a need to understand the mechanisms and processes that underpin microbial community assembly, and which determine the spatial and temporal distributions of different microbial taxa [29, 30]. A plethora of potential ecological processes that could explain such patterns have been distilled into the influence and interplay between four basic processes; selection, dispersal, drift, and speciation [14]. This simplified framework of ecological processes has been strongly advocated as informative for microbial ecology [29, 30]. In brief, dispersal limitation, or selection, results from biotic and abiotic pressures causing minimal exchange of organisms between communities. Homogenizing dispersal is the degree to which individuals of species move between and successfully establish in local communities. Drift results from stochastic changes in population sizes, and speciation is the evolution of new species [14, 25].

Here the Raup and Crick probability-based index of similarity (*S*_RC_) was used to test to what extent ecological processes (homogenizing dispersal, dispersal limitation/selection, and drift) accounted for temporal turnover within each tree-hole community [24]. This index has long been used to examine the influence of deterministic and stochastic factors on community assembly e.g., [8, 24, 31]. It has been noted that some ecological processes of community assembly [14] can map easily onto this deterministic-stochastic framework [32]. To that end, we used *S*_RC_ values that were deterministically similar (*S*_RC_ ≥ 0.95) or dissimilar (*S*_RC_ ≤ −0.95) than expected by chance to infer the deterministic processes of homogenising dispersal and dispersal limitation (selection), respectively [32]. In addition, we used *S*_RC_ values that indicated similarity no greater than expected by chance (*S*_RC_ > −0.95 and <0.95) to infer the stochastic process of drift [24, 25].

**Fig. 4:**
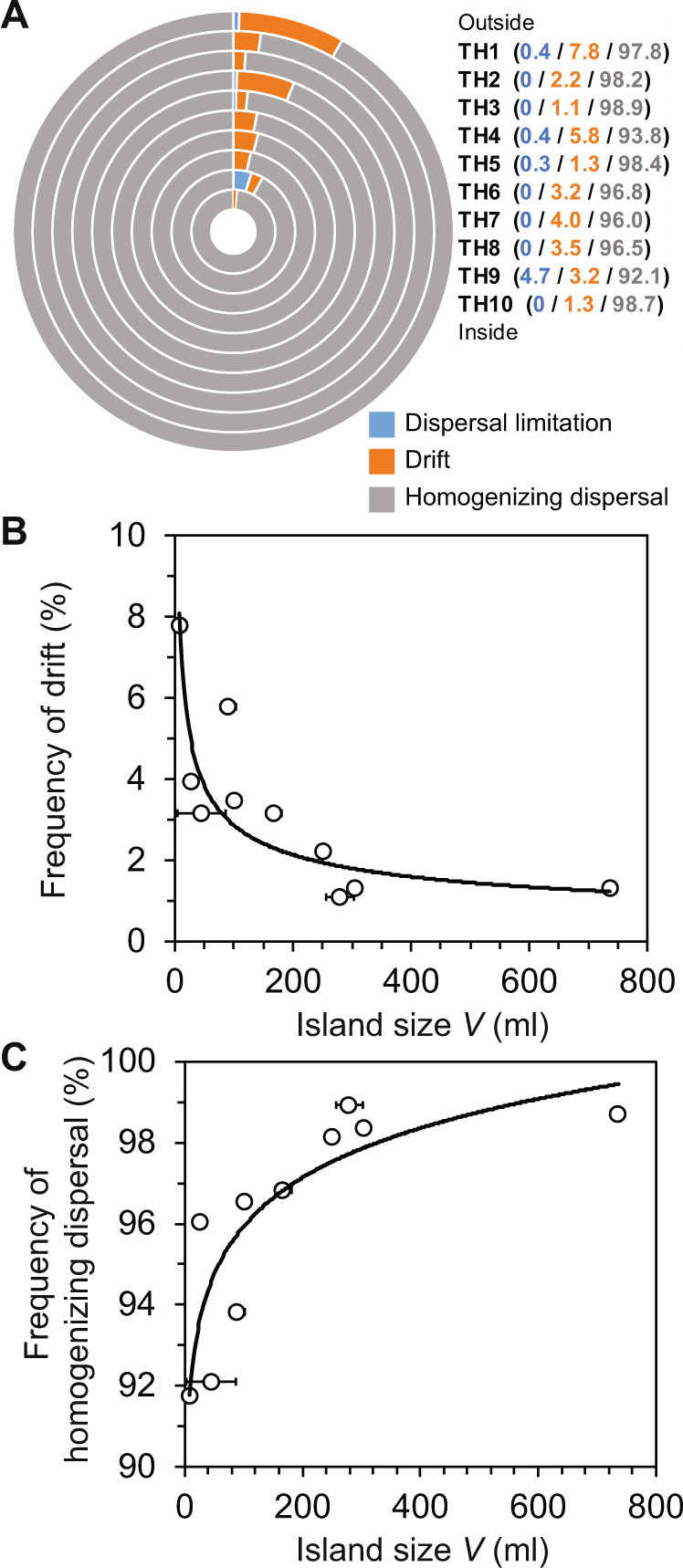
Ecological process relationships with island size and taxa turnover. (**A**) Percentage frequency of Raup and Crick probability-based index pairwise values, for each tree-hole, assigned to dispersal limitation, drift, and homogenizing dispersal. Tree-holes (TH) are presented from TH1, as the outmost circle, through to TH10, as the innermost. Also given for each tree-hole are the percentage frequency values accounted for by each of the ecological processes. **B, C** are the relationships between island size (volume, *V*) with (**B**) drift and homogenizing dispersal, respectively. Regression statistics: (**B**) *R*^2^ = 0.71, *F*_1,8_ = 20.1, *P* = 0.0002; and (**C**) *R*^2^ = 0.72, *F*_1,8_ = 20.5, *P* = 0.0002. The relationship between *V* and dispersal limitation was non-significant and is not shown: *R*^2^ = 0.04, *F*_1,8_ = 0.4, *P* = 0.560. Horizontal error bars represent standard deviation of the mean of *V* over census time.

Strikingly, similar patterns of pairwise *S*_RC_ frequencies were observed within all of the tree-hole communities (Fig. 4A). Communities were mainly characterized by the deterministic process of homogenizing dispersal (mean *S*_RC_ = 96.1, SD ± 2.5%), and to a lesser extent by drift (3.3 ± 2.0%) and then dispersal limitation (0.6 ± 1.4%). Speciation is not explicitly accounted for using the *S*_RC_ index, but can cause differences in diversity among sets of communities that do not exchange through dispersal [14]. Therefore, speciation should have negligible influence within a set of communities where individuals disperse among local communities within a metacommunity, as was the case in this study [25]. Moreover, here speciation should be negligible given the survey’s timeframe and the method used to define taxa (i.e., 16 S rRNA amplicon sequencing and grouping in 97% OTUs).

The low frequency of dispersal limitation and high homogenizing dispersal indicated that island isolation was unimportant, as postulated earlier (Fig. 4A). Therefore, turnover was related to island size and not island isolation within the study system. We posit that the large degree of homogenizing dispersal was due to the negligible effect of isolation from the immigration sources and the relatively small distances between islands in this tree-hole archipelago, with the minimum and maximum distance between any two adjoining tree-holes was 12.5 and 217 cm, respectively (mean 59.7 ± 62.9 cm). In support of this view, a recent study experimentally demonstrated dispersal homogenized bacterial communities via immigration, and not through weakening selection, within a metacommunity at small spatial scales [33].

To examine the influence of island size further, we explored associations with ecological processes (Fig. 4B&C). We found a significant inverse relationship between drift and island size (Fig. 4B) and, conversely, a significant positive relationship between homogenizing dispersal and island size (Fig. 4C). Theory predicts that species turnover will be higher on smaller islands than on larger islands, as was the case in the current study (Fig. 3C), [1, 4, 26]. This can be due to islands of decreasing size having an increased probability of chance events [1, 4]. Such stochastic events include death, reproduction, and migration, which underpin the stochastic-based process of drift [14, 24]. Hence, we posit this may explain the observation of drift significantly increasing with decreasing island size in the current study. Moreover, in the same spirit, others have proposed that with smaller island sizes, there are inherent smaller population sizes, thus making it harder for homogenizing dispersal to effectively increase population size and counterbalance drift [34, 35]. As such, this could help better explain observed patterns and highlight dispersal and drift are inversely linked [35]. That could be determined in future work, which manipulates dispersal rates, population sizes, and island sizes in experimental tree-holes.

Here we provide, for the first time, determination of the underlying mechanisms and processes that can underpin STRs (regardless of STR construction approach), and hence temporal turnover, within island-based bacterial communities. We found that island size was important in influencing turnover within the bacterial communities studied. Moreover, homogenizing dispersal was the dominant ecological process driving community turnover within the study system. That observation, coupled with a negligible influence of dispersal limitation, was also the result of the lack of island isolation from the immigration source in this study system. Future studies could determine which processes underpin temporal turnover when variation in island isolation is experimentally controlled and manipulated. While the SAR has been pivotal in the development of ecological models and theories, we are optimistic that the findings from the current study will provide new impetus in realizing the potential of its understudied and less understood temporal analogue, the STR.

## Supplementary information

Supplemental material
