# Supplementary material for: Bacterial communities in larger islands have reduced temporal turnover
Source: ISME J. 2021 May 3;15(10):2947–55. doi: 10.1038/s41396-021-00976-0 (PMC8443627; doi:10.1038/s41396-021-00976-0)
Supplement: Supplementary file 1 — Supplemental material [file 41396_2021_976_MOESM1_ESM.pdf]

## Supplemental materials

# Bacterial communities in larger islands have reduced temporal turnover

Damian W. Rivett[1], Shorok B. Mombrikotb[2], Hyun S. Gweon[3], Thomas Bell[2], Christopher van der Gast[4*]

[1]Department of Natural Sciences, Manchester Metropolitan University, Manchester, UK

[2]Department of Life Sciences, Imperial College London, Ascot, UK

[3]School of Biological Sciences, University of Reading, Reading, UK

[4]Department of Life Sciences, Manchester Metropolitan University, Manchester, UK

*Correspondence: Prof Chris van der Gast, Manchester Metropolitan University, Department of Life Sciences, Chester Street, John Dalton Building, Manchester, M1 5GD, UK.

E-mail: C.vanderGast@mmu.ac.uk

### Bioinformatics code from Sequence analyses

# All processing steps have been incorporated into a set of reproducible scripts/utilities uploaded in https://github.com/hsgweon/sgtoolkit/tree/master/sgtoolkit

Having cloned and installed this utilities as instructed on the page, we used the following commands:

```
sgtk_make_file_pairs_list.py -i rawdata_dir

sgtk_prepseqs.py -i rawdata_dir -o sgtk_prepseqs -l file_pairs_list.txt

vsearch --sortbylength prepseqs/prepped.fasta --minseqlength 300 --output
prepseqs/prepped_lf_sorted.fasta --fasta_width 0

bbduk.sh -Xmx1g in=prepseqs/prepped_lf_sorted.fasta out=prepseqs/all_prepped.fasta
outm=prepseqs/prepped_contaminated.fasta ref=/raid2/prjscratch/BIOINFORMATICSDB/phix.fasta k=31
hdist=1 stats= prepseqs/bbduk_stats.txt overwrite=true

sgtk_processseqs.py -i prepseqs/all_prepped.fasta -o sgtk_processseqs --region 16S -v -t 10 -r
```

**Table S1** Species-Time Relationship (STR) power function regression statistics.

| Tree-hole | Method | Time-series length (days) | Slope *w* | Intercept *c* | Degrees of freedom | *F*-statistic | $R^2$ | *P* |
|---|---|---|---|---|---|---|---|---|
| TH1 | EPW | 364 | 0.0983 | 307.9 | 1,20 | 61.92 | 0.76 | <0.0001 |
| TH2 | EPW | 364 | 0.0576 | 367.4 | 1,15 | 14.90 | 0.50 | 0.001 |
| TH3 | EPW | 364 | 0.0763 | 481.9 | 1,22 | 70.97 | 0.76 | <0.0001 |
| TH4 | EPW | 364 | 0.1064 | 366.1 | 1,22 | 55.74 | 0.72 | <0.0001 |
| TH5 | EPW | 280 | 0.0565 | 492.6 | 1,16 | 159.2 | 0.91 | <0.0001 |
| TH6 | EPW | 350 | 0.0694 | 395.1 | 1,22 | 41.23 | 0.66 | <0.0001 |
| TH7 | EPW | 334 | 0.1232 | 270.3 | 1,22 | 79.36 | 0.79 | <0.0001 |
| TH8 | EPW | 364 | 0.1183 | 299.1 | 1,20 | 88.56 | 0.82 | <0.0001 |
| TH9 | EPW | 334 | 0.1284 | 269.1 | 1,21 | 31.32 | 0.60 | <0.0001 |
| TH10 | EPW | 280 | 0.0476 | 486.2 | 1,16 | 52.86 | 0.77 | <0.0001 |
| TH1 | CMW | 364 | 0.1278 | 302.1 | 1,20 | 94.66 | 0.83 | <0.0001 |
| TH2 | CMW | 364 | 0.0754 | 386.8 | 1,15 | 65.07 | 0.81 | <0.0001 |
| TH3 | CMW | 364 | 0.0919 | 487.7 | 1,22 | 143.37 | 0.87 | <0.0001 |
| TH4 | CMW | 364 | 0.1253 | 369.6 | 1,22 | 91.69 | 0.81 | <0.0001 |
| TH5 | CMW | 280 | 0.0793 | 492.0 | 1,16 | 335.6 | 0.95 | <0.0001 |
| TH6 | CMW | 350 | 0.0954 | 397.3 | 1,22 | 110.71 | 0.84 | <0.0001 |
| TH7 | CMW | 334 | 0.1495 | 271.0 | 1,22 | 140.29 | 0.87 | <0.0001 |
| TH8 | CMW | 364 | 0.1466 | 290.2 | 1,20 | 117.1 | 0.85 | <0.0001 |
| TH9 | CMW | 334 | 0.1449 | 281.3 | 1,21 | 58.71 | 0.74 | <0.0001 |
| TH10 | CMW | 280 | 0.0732 | 487.6 | 1,16 | 179.64 | 0.92 | <0.0001 |
| TH1 | MW | 364 | 0.3094 | 198.9 | 1,20 | 40.9 | 0.67 | <0.0001 |
| TH2 | MW | 364 | 0.2356 | 288.8 | 1,15 | 37.61 | 0.72 | <0.0001 |
| TH3 | MW | 364 | 0.2818 | 319.2 | 1,22 | 52.31 | 0.7 | <0.0001 |
| TH4 | MW | 364 | 0.3144 | 242.3 | 1,22 | 49.37 | 0.69 | <0.0001 |
| TH5 | MW | 280 | 0.2237 | 380.8 | 1,16 | 60.48 | 0.79 | <0.0001 |
| TH6 | MW | 350 | 0.2894 | 246.1 | 1,22 | 46.37 | 0.68 | <0.0001 |
| TH7 | MW | 334 | 0.3498 | 171.1 | 1,22 | 54.98 | 0.72 | <0.0001 |
| TH8 | MW | 364 | 0.3187 | 197.9 | 1,20 | 46.4 | 0.70 | <0.0001 |
| TH9 | MW | 334 | 0.3402 | 185.1 | 1,21 | 50.44 | 0.71 | <0.0001 |
| TH10 | MW | 280 | 0.2232 | 369.9 | 1,16 | 39.76 | 0.71 | <0.0001 |

STR construction method: EPW, Every Possible Window; CMW, Cumulative Moving Window; and MW, Moving Window.

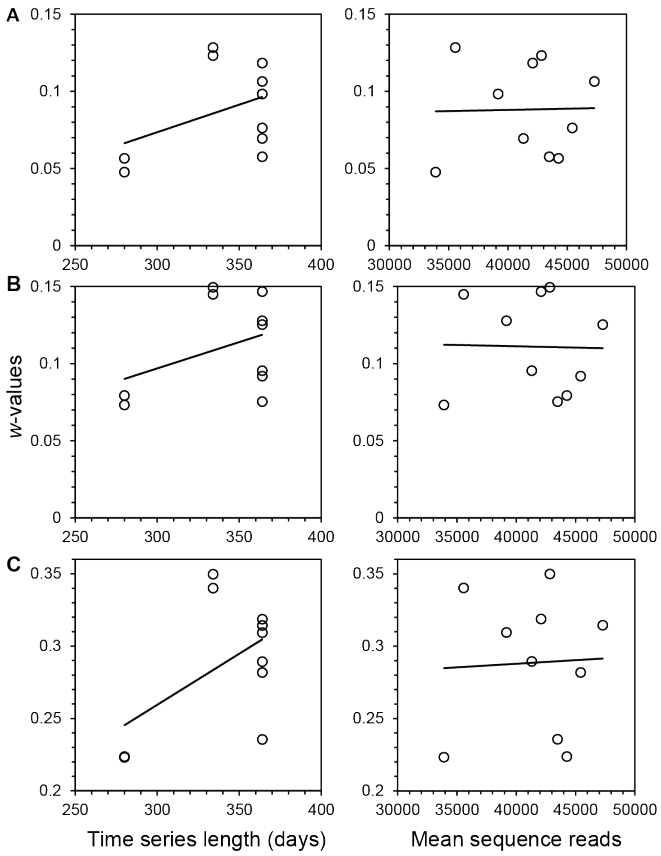


**Figure S1** Testing for STR scaling exponent (*w*) relationships with Time series length and Sequencing depth. Values of *w* derived from the (A) every possible windows (EPW), (B) cumulative moving window (CMW), and (C) moving window (MW) approaches. Regression statistics: (A) EPW with time, $R^2$ = 0.16, $F_{1,18}$ = 1.58, $P$ = 0.245. EPW with mean sequence depth, $R^2$ = 0.001, $F_{1,18}$ = 0.001, $P$ = 0.953. (B) CMW with time, $R^2$ = 0.14, $F_{1,18}$ = 1.34, $P$ = 0.281. CMW with mean sequence depth, $R^2$ = 0.01, $F_{1,18}$ = 0.001, $P$ = 0.948. (C) MW with time, $R^2$ = 0.28, $F_{1,18}$ = 3.12, $P$ = 0.115. MW with mean sequence depth, $R^2$ = 0.002, $F_{1,18}$ = 0.002, $P$ = 0.902.
